# Investigating the gut microbiota in advanced heart failure and cardiac cachexia

**DOI:** 10.1080/29933935.2026.2670244

**Published:** 2026-06-07

**Authors:** Mansimran Singh Dulay, Despoina Chrysostomou, Monica Campos, Marko Storch, Lauren A. Roberts, Damanpreet Singh Dev, Nahal Raza, Ramey Assaf, Tricia Tan, Michael Marber, Rakesh Sharma, Thomas Lüscher, Julian R. Marchesi, Owais Dar

**Affiliations:** a King’s College London School of Cardiovascular Medicine and Sciences, London, UK; b Department of Advanced Heart Failure, Transplant and Mechanical Circulatory Support, Harefield Hospital, Royal Brompton and Harefield Hospitals, part of Guy’s and St Thomas’ NHS Foundation Trust, London, UK; c Division of Digestive Diseases, Department of Metabolism, Digestion and Reproduction, Imperial College London, London, UK; d London Biofoundry, Imperial College Translation & Innovation Hub, London, UK; e Department of Infectious Disease, Imperial College London, London, UK; f Department of Metabolism, Digestion and Reproduction, Imperial College London, London, UK; g National Heart and Lung Institute, Imperial College London, London, UK; h Department of Cardiology, Royal Brompton and Harefield Hospitals, part of Guy’s and St Thomas’ NHS Foundation Trust, London, UK

**Keywords:** Advanced heart failure, gut microbiota, cardiac cachexia, unintentional weight loss, prognosis

## Abstract

Cardiac cachexia (CC) is associated with advanced heart failure (AHF), characterized by unintentional weight loss (UWL) of fat/muscle. It is exacerbated by right ventricular systolic dysfunction (RVSD). The potential pathogenic role of gut microbiota (GM) changes has not been investigated in CC. We aimed to explore this. Patients with AHF with or without CC/UWL, stable chronic heart failure (HF), and healthy controls (HCs) were recruited following national ethical approval. Fecal bacterial DNA was extracted, quantified, and 16S rRNA gene sequencing was performed. GM composition, alpha, and beta diversity were compared between CC/UWL-AHF and the rest of the cohort (ROC). The secondary analyses compared AHF, HF, and HCs, and patients with and without RVSD. Sixty-seven patients returned samples, including 14 with CC/UWL-AHF. No taxonomic differences were observed between CC/UWL-AHF and ROC. A weak trend toward compositional differences was observed (beta diversity R²  =  0.016, *p*  =  0.071). No differences were observed in RVSD. Numerous significant GM alterations were observed across the HF spectrum, including changes to *Streptococcus spp.*, *Alistipes*, and *Bacteroides*. CC/UWL-AHF may be associated with subtle GM compositional changes. Larger studies are required to investigate this further.

## Introduction

Heart Failure (HF) is a clinical syndrome, with an underlying diagnosis of left or right ventricular dysfunction coupled with clinical signs and symptoms of fluid overload.^1^ Progression of HF can lead to the development of an end-stage phenotype, termed advanced heart failure (AHF), with a correspondingly very poor prognosis.^2^ A recognized indicator of AHF in international guidance is cardiac cachexia (CC).^3^ Cardiac cachexia is currently defined as a metabolic syndrome with >5% unintentional weight loss (UWL) of fat and or muscle as a proportion of total body weight, or a low body mass index <18 kg/m^2^, as major criteria, alongside several minor criteria incorporating factors such as low muscle mass and strength, poor appetite, fatigue, and biochemical derangements.^4^ Important pathogenic causes behind CC formation include a heightened overall pro-inflammatory state contributing to proteolysis and lipolysis, alongside reduced appetite through alterations to physiological gut hormone signaling.^5^ ^,^ ^6^

Studies have identified that disproportionate right ventricular systolic dysfunction (RVSD) may have an important pathophysiological role in the development of CC, resulting in exacerbated gut oedema with associated poorer appetite and earlier satiety.^7^ Moreover, intestinal congestion has been linked with increased gut permeability through disrupting the orderly functioning of the blood gut barrier (BGB), with subsequent exacerbation of pro-inflammatory signaling secondary to bacterial translocation.^8^

The gut microbiota (GM), a descriptive term for the microorganisms within the gastrointestinal tract, have numerous important physiological functions not limited to: processing host and dietary derived nutrients into metabolites, maintaining the BGB itself and absorption of vitamins.^9^ Increasing emphasis is being placed on understanding the role of the GM in varying disease states, with alterations to a healthy GM (‘dysbiosis’) linked to a range of inflammatory and autoimmune pathologies.^10^ In HF, prior studies have identified both reduction in overall GM bacterial diversity and decrease in the abundance of specific bacterial taxonomic levels.^11^ Whether alterations to the healthy GM have a pathogenic role in CC specifically, however, has not yet been investigated. Given the importance of CC in identifying AHF and therefore patients with poorer prognostic outcomes, understanding potentially modifiable risk factors remains pertinent; in other disease states, modifying the GM through faecal microbial transplantation is a promising area of growing interest.^12^

The primary objective of this study was to assess for changes in the composition of the GM in AHF patients with CC, ultimately to ascertain novel pathways of disease pathogenesis. In addition, the secondary objective of this study aimed to analyze any GM compositional differences between those patients with AHF and HF, in those with and without a diagnosis of HF, and in those HF patients with and without RVSD.

## Materials and methods

### Study population, sample collection, and ethical approval

Inpatients and outpatients were recruited from Harefield Hospital (Royal Brompton and Harefield Hospitals, part of Guy’s and St Thomas’ NHS Foundation Trust) following written informed consent between August 2024 and January 2025. Healthy control subjects were also recruited during this period. This study received full United Kingdom (UK) Health Regulatory Authority approval (Research Ethical Council 24/PR/0437).

Patients recruited were divided into the following groups: CC/UWL and AHF, AHF in the absence of CC/UWL, stable chronic HF, and healthy controls (HCs). Advanced heart failure was defined using the 2022 American Heart Association “I NEED HELP” criteria^3^ – therefore, if a patient with known acute or chronic HF met the ≥1 criterion, they were categorized as having AHF accordingly. Healthy controls had no known co-morbidities, nor were not taking any regular medications. No patient had received antibiotics within the preceding 4 weeks to enrolment, and unless receiving intravenous inotropic agents or diuretics, they had not undergone any changes to their usual pharmacological regime. Enrolled patients had relevant baseline demographics, and clinical indices recorded.

The AHF cohort was assessed for CC using the current 2008 consensus definition.^4^ At baseline, patients were weighed (dry weight), had muscle strength readings taken using hand grip strength, measures of fat and muscle mass were taken via mid-arm circumference measurements, and questionnaires were used to assess fatigue, appetite, and quality of life. Some patients demonstrated significant UWL (>5%) in the absence of meeting the full CC consensus definition and were therefore included in the CC/UWL-AHF patient group. Any recorded UWL was oedema-free within the preceding 12 months; patients with weight loss due to reversible causes such as thyroid or gastrointestinal diseases or cancer were not included in the CC/UWL-AHF group. The enrolled patients were subsequently provided with a stool collection kit (OMNIgene.GUT, DNA Genotek, Ottawa), providing sample stability for up to 60 d post-collection at room temperature. The samples were returned within 48 h of collection and frozen at −80°C until analysis.

#### Sample analysis: bacterial DNA extraction and quantification

Sample analysis was undertaken at the Imperial College London Division of Digestive Diseases, St. Mary’s Hospital, London. DNA extraction was performed using the DNeasy 96 PowerSoil Pro QIAcube HT Kit (Qiagen, Germany) with the QIAcube HT automated platform to standardize handling. DNA extraction was performed as recommended by the manufacturer with the addition of a heating step (65 °C 10 min) prior to homogenization by beating using the Bullet blender Storm (Thistle Scientific, USA) for 3 min at speed 8. DNA was eluted in 10 mM Tris-Cl buffer (pH 8.5). The DNA concentration was measured using the Qubit™ 4 Fluorometer (ThermoFisher Scientific, Massachusetts, United States of America [USA]) with a 1X dsDNA broad range assay kit as per the manufacturer’s instructions. DNA samples were stored at −20 °C until further analysis.

Total bacterial 16S rRNA gene copies were quantified using the BactQUANT qPCR assay as previously described.^13^ ^,^ ^14^ In brief, 5 µL of extracted genomic DNA was added to a 25 µL reaction containing KAPA HiFi HotStart ReadyMix (Roche, Penzberg, Germany) supplemented with ROX reference dye (1:50 dilution, Biotium, USA), 1.8 µM BactQuant forward and reverse primers, and 225 nM MGB probe (Applied Biosystems, USA). Amplification and real-time fluorescence detection were carried out using a StepOnePlus Real-Time PCR System (Applied Biosystems, USA). Cycling conditions were applied as described previously.^14^ Standard curves were generated from serial dilutions of *Escherichia coli* genomic DNA, and amplification was performed on a StepOnePlus Real-Time PCR System (Applied Biosystems, ThermoFischer Scientific, USA). The total bacterial load was expressed as the 16S rRNA gene copy number per µL of DNA extract.

#### Long-read 16S rRNA gene sequencing

Full-length 16S rRNA gene libraries were prepared by amplifying the V1–V9 region using Pacific Biosciences (PacBio) forward (5′ GCATC/barcode/AGRGTTYGATYMTGGCTCAG 3′) and reverse (5′ GCATC/barcode/RGYTACCTTGTTACGACTT 3′) primers. The DNA concentrations were normalized to 2 ng/µL prior to amplification using KAPA HiFi HS ReadyMix (Roche Diagnostics, UK). Each sample was assigned unique PacBio barcode sequences on both the forward and reverse primers to enable multiplexed sequencing. Barcoded amplicons were arrayed into PCR plates and sequenced on the Sequel IIe system (PacBio, USA) using one SMRT Cell 8M, yielding an average of 13,315 reads per sample.

#### Statistical analyses

Baseline characteristics were compared between the CC/UWL-AHF group and rest of cohort (ROC). Categorical variables were expressed as raw number and percentage, with between-group comparisons made using Chi- square or Fischer’s exact test. Numerical variables were assessed for normality and mean, or median, values were compared between groups using independent 2-sample t-test or a Wilcoxon rank-sum test, respectively.

Raw sequencing reads (FASTQ format) were processed using the DADA2 pipeline (Version 1.22.0) within R (Version 4.2.3). All files have been submitted to the EMBL-EBI European Nucleotide Archive (ENA) under accession number PRJEB105609. The initial filtering steps included removal of the first 19 bases corresponding to primer sequences, trimming of reads with quality scores below 30, and exclusion of reads with more than two expected errors or unique sequences. Default DADA2 parameters were used for dereplication, denoising, merging, chimera removal, and inference of amplicon sequence variants (ASVs). Sequence inference was performed in pseudo-pooled mode. Taxonomic assignment of ASVs was conducted using the SILVA reference database (Version 138.1).

The relative abundances of ASVs underwent subsequent ecological analyses with between-group comparisons, following centred log-ratio transformation. The primary analysis compared the CC-UWL/AHF group with the ROC, with secondary analyses comparing the AHF, HF and HC groups and patients with and without RVSD (defined as an RV fractional area change  <35% on baseline transthoracic echocardiography). Alpha diversity was assessed using Shannon’s index, and beta diversity using principal coordinate analysis (PCoA) based on Bray‒Curtis distances, with PERMANOVA analyses performed based upon 999 permutations. Between-group taxa differences were assessed using the Kruskal–Wallis (KW) rank sum test, followed by post-hoc Dunn tests. Analyses underwent multiple testing correction using the Benjamini‒Hochberg method to control for a 5% false discovery rate, and were deemed significant if adjusted *p* ≤ 0.05. Statistical analyses were conducted using SPSS (v31.0, IBM, USA), R (v4.4.2) via *phyloseq and vegan* packages and MicrobiomeAnalyst 2.0.^15-18^

## Results

A total of 67 patients returned fecal samples within the study period, of whom 14 had CC/UWL-AHF. Of the remaining 53 patients, 33 had AHF in the absence of CC, 10 had stable chronic HF, and 10 were healthy controls. Of those patients with HF, 32 had RVSD. Table 1 summarizes baseline characteristic differences between those with and without CC/UWL-AHF. Overall, the CC/UWL-AHF group was more likely to have numerous baseline indicators of poor overall prognosis, not limited to a more advanced New York Heart Association class, a lower body mass index, higher natriuretic peptide levels, and poorer left ventricular ejection function (Table 1).

**Table 1.** Baseline characteristics.

	CC/UWL-AHF *n* = 14	Rest of cohort *n* = 53	*p* value
Gender	Male 11/14 (79%) Female 3/14 (21%)	Male 36/53 (68%) Female 17/53 (32%)	*p* = 0.528
Age (years)	53.64+/***−*** 6.73	53.00+/***−*** 20.00	*p* = 0.537
Ethnicity	Afro-Caribbean 1/14 (7%) East Asian 1/14 (7%) Mixed race 1/14 (7%) South Asian 1/14 (7%) Caucasian 10/14 (71%)	Afro-Caribbean 3/53 (6%) East Asian 1/53 (2%) Mixed race 0/53 (0%) South Asian 4/53 (8%) Caucasian 45/53 (85%)	*p* = 0.277
HF aetiology	ACHD 0/14 (0%) ARVC 1/14 (7%) DCM 6/14 (43%) HCM/restrictive 0/14 (0%) ICM 6/14 (43%) Mixed ICM/DCM 1/14 (7%) Healthy control 0/14 (0%)	ACHD 1/53 (2%) ARVC 2/53 (4%) DCM 25/53 (47%) HCM/restrictive 4/53 (8%) ICM 11/53 (21%) Mixed ICM/DCM 0/53 (0%) Healthy control 10/53 (19%)	*p* = 0.116
***NYHA class***	***I 0/14 (0%)*** ***II 3/14 (21%)*** ***III/IV 11/14 (79%)***	***I 12/43 (28%)*** ***II 16/43 (37%)*** ***III/IV 15/53 (35%)***	***p = 0.010***
***BMI (kg/m*** ^***2***^ ***)***	***21.16+/−1.94***	***28.18+/−4.08***	***p < 0.001***
***Weight change (%)***	***−12.83+/−7.76***	***+0.41+/−4.68***	***p < 0.001***
***HR (bpm)***	***79.00+/−23.00***	***68.19+/−14.85***	***p = 0.002***
***SBP (mmHg)***	***94.50+/−15.00***	***102.02+/−15.44***	***p = 0.009***
Smoking history	6/14 (43%)	13/53 (25%)	*p* = 0.196
Asthma/COPD	2/14 (14%)	5/53 (9%)	*p* = 0.630
HTN	1/14 (7%)	8/53 (15%)	*p* = 0.672
CKD	3/14 (21%)	7/53 (13%)	*p* = 0.425
Previous malignancy	1/14 (7%)	1/53 (2%)	*p* = 0.377
Thyroid disease	0/14 (0%)	8/53 (15%)	*p* = 0.189
TIIDM	1/14 (7%)	8/53 (15%)	*p* = 0.672
IHD	6/14 (43%)	10/53 (19%)	*p* = 0.082
Hypercholesterolaemia	8/14 (57%)	15/53 (28%)	*p* = 0.060
Current beta blocker	11/14 (79%)	42/53 (79%)	*p* = 0.100
Current ARNI	9/14 (64%)	33/53 (62%)	*p* = 0.755
Current ACEi/ARB	4/14 (29%)	7/53 (14%)	*p* = 0.227
Current MRA	11/14 (79%)	38/53 (72%)	*p* = 0.743
Current SGLT-2i	12/14 (86%)	38/53 (72%)	*p* = 0.491
Current PO loop diuretic	8/14 (57%)	26/53 (49%)	*p* = 0.765
Current IV diuretic	2/14 (14%)	3/53 (6%)	*p* = 0.278
Current IV inotropes	4/14 (29%)	5/53 (9%)	*p* = 0.083
***NT-proBNP (pg/l)***	***2363.50 + /−3413.00***	***1065.00 + /−2556.00***	***p = 0.025***
pVO2 (ml/kg/min)	17.45 + /−5.57	17.45 + /−9.10	*p* = 0.813
Predicted pVO2 (%)	50.00 + /−27.00	70.34 + /−25.54	*p* = 0.141
***LVEF (%)***	***24.50 + /−9.27***	***28.00 + /−23.00***	***p = 0.044***
RAP (mmHg)	10.86 + /−4.78	8.50 + /−4.00	*p* = 0.502
PCWP (mmHg)	22.14 + /−9.21	19.33 + /−6.28	*p* = 0.387
CO (l/min)	3.41 + /−1.59	3.45 + /−1.00	*p* = 0.303
CI (l/min/m^2^)	1.72 + /−0.39	1.82 + /−0.71	*p* = 0.431
PVR (WU/m^2^)	2.78 + /−0.90	2.75 + /−1.23	*p* = 0.946

*Baseline characteristic data with comparisons between the CC/UWL-AHF group and rest of cohort. Abbreviations: CC—cardiac cachexia, UWL—unintentional weight loss, AHF—advanced heart failure, ACHD—adult congenital heart disease, ARVC—arrhythmogenic right ventricular cardiomyopathy, DCM—dilated cardiomyopathy, HCM—hypertrophic cardiomyopathy, ICM—ischaemic cardiomyopathy, NYHA—New York Heart Association, BMI—body mass index, HR—heart rate, SBP—systolic blood pressure, COPD—chronic obstructive pulmonary disease, HTN—hypertension, CKD—chronic kidney disease, TIIDM—type II diabetes mellitus, ARNI—angiotensin receptor neprilysin inhibitor, ACEi—angiotensin converting enzyme inhibitor, ARB—angiotensin receptor blocker, MRA—mineralocorticoid receptor antagonist, SGLT-2i sodium-glucose cotransporter 2 inhibitor, PO—per os, IV—intravenous, pVO2—peak oxygen uptake, LVEF—left ventricular ejection fraction, RAP—right atrial pressure, PCWP—pulmonary capillary wedge pressure, CO—cardiac output, CI—cardiac index, PVR—pulmonary vascular resistance.*

The bold values simply reflect those that met statistical significance.

**Figure 1.**
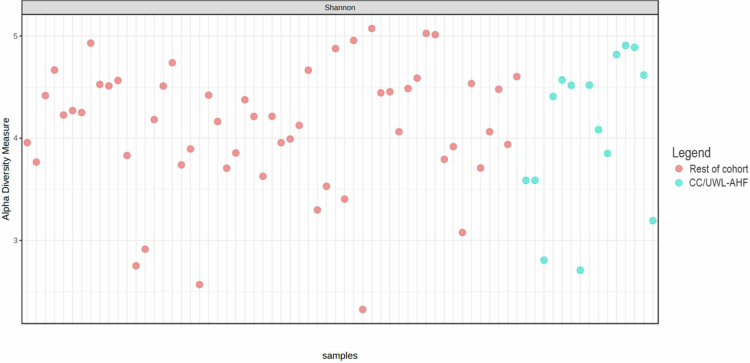
Alpha diversity plot (measured by Shannon’s index) comparing CC/UWL-AHF group with ROC.

**Figure 2.**
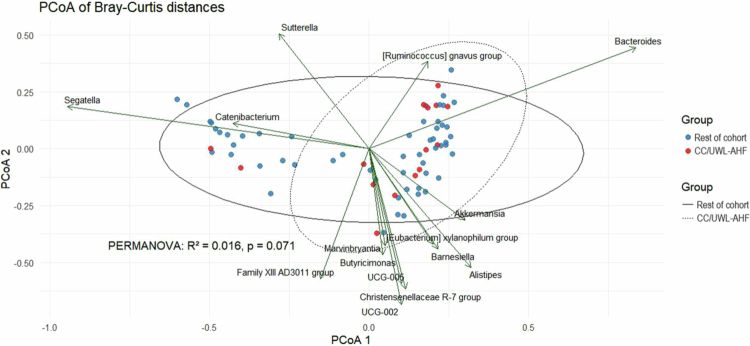
Beta diversity PCoA plot comparing CC/UWL-AHF and ROC groups. The 15 most significant families implicated in compositional differences have been plotted in addition.

No significant differences were noted with regards to alpha diversity between the CC/UWL-AHF and ROC, with a Shannon index of 4.79 (CC/UWL-AHF) versus 4.71 (ROC), *p*  =  0.84. (Figure 1). This remained the case with regard to the secondary analyses performed when comparing the AHF, HF, and HC groups, and patients with and without RVSD. Upon assessing beta diversity, a borderline significant weak trend towards overall community composition difference was noted between the CC/UWL-AHF and ROC groups (R^2^  =  0.016, *p*  =  0.071), with the 15 bacterial families contributing most significantly towards community separation demonstrated in Figure 2. Secondary analyses did not reveal any differences in beta diversity between AHF, HF, and HC groups, or patients with and without RVSD.

KW testing did not reveal any GM compositional changes in the CC/UWL-AHF group when compared to ROC, or in those with or without RVSD. A number of significant GM changes were observed when analyzing the AHF, HF, and HC groups, as summarized in Table 2. *Streptococcus* species (spp) were enriched in the AHF group versus HCs, Alistipes in the AHF when compared to HF, *Family XIII AD3011 group* in the AHF when compared to both HF and HC groups, *Butyricimonas* and *Bacteroides* in the HF versus HC group, and *Parasutterella* in the AHF and HF versus HCs (all adjusted *p*  <  0.05).

**Table 2.** Summary of gut microbiota changes in patients with AHF, HF and HCs.

Taxon	Comparison	Adjusted *P* value	Higher in Group	Phylum	Family	Species
*Streptococcus* (Streptococcaceae)	AHF vs HCs	0.00331	AHF	Bacillota	Streptococcaceae	*Streptococcus salivarius*
*Streptococcus* (Streptococcaceae)	AHF vs HCs	0.01250	AHF	Bacillota	Streptococcaceae	*Streptococcus oralis*
*Alistipes* (Rikenellaceae)	AHF vs HF	0.00814	AHF	Bacteroidota	Rikenellaceae	*Alistipes indistinctus*
Family XIII AD3011 group (Anaerovoracaceae)	AHF vs HF	0.00977	AHF	Bacillota	Anaerovoracaceae	—
Family XIII AD3011 group (Anaerovoracaceae)	AHF vs HCs	0.01329	HF	Bacillota	Anaerovoracaceae	—
*Butyricimonas* (Marinifilaceae)	HF vs HCs	0.01240	HF	Bacteroidota	Marinifilaceae	—
*Bacteroides* (Bacteroidaceae)	HF vs HCs	0.01078	HCs	Bacteroidota	Bacteroidaceae	*Bacteroides uniformis*
*Parasutterella* (Sutterellaceae)	HF vs HCs	0.01176	HCs	Pseudomonadota	Sutterellaceae	—
*Parasutterella* (Sutterellaceae)	AHF vs HCs	0.01793	HCs	Pseudomonadota	Sutterellaceae	—

*Gut microbiota changes based upon Kruskal–Wallis (KW) rank sum and post-hoc Dunn testing. Abbreviations: AHF—advanced heart failure, HF—heart failure, and HCs—healthy controls.*

## Discussion

To our knowledge, this is the first study in which the GM composition of patients with AHF and CC/UWL specifically has been investigated. Our beta diversity analysis showed a trend towards statistical significance, demonstrating that CC/UWL-AHF status explained ~1.6% of the variance in microbiome structure when compared to patients without (*p*  =  0.071). (Figure 2) Our findings may support the notion that AHF patients with CC/UWL harbor modestly distinct microbial community profiles, and further research is prudent to ascertain if such changes are responsible for disease pathogenesis. The presence of RVSD did not alter the GM composition. Additionally, our secondary analyses yielded a number of GM compositional changes within the spectrum of AHF and HF (Table 2).

Whilst there is a lack of literature pertaining to the composition and potential pathogenic role of the GM in CC, changes in general HF populations have been described. It can be hypothesized that chronic HF exhibits alterations in the GM through gut hypoperfusion.^8^ Sandek and colleagues demonstrated a correlation between increased bowel wall oedema with subsequent permeability across the blood-gut barrier in chronic HF patients when compared to healthy controls.^19^ Numerous studies have demonstrated specific alterations to the GM in HF, which may exacerbate chronic inflammation alongside the production of important metabolites; importantly, a large proportion of these studies noted a reduction in alpha diversity in HF, with correspondingly elevated levels of both systemic inflammation alongside inappropriate circularization of gut-derived pro-inflammatory endotoxins.^8^ ^,^ ^20^

Changes to certain bacterial phyla have been implicated in HF alongside an exacerbated pro-inflammatory response. Mayerhofer and co-workers noted a reduction in *Firmicutes (Bacillota)* and elevated *Bacteroides* phyla ratio in HF patients in the context of low-fibre diets, with an exaggeration of this ratio in those who reached an endpoint of mortality or cardiac transplantation.^21^ Importantly, Yuzefpolskaya and colleagues demonstrated that reductions to *Lachnospiraceae* or *Ruminococcaceae* (pertaining to the *Firmicutes* phylum) were present with worsening HF phenotype, alongside elevated pro-inflammatory endotoxins and overall reduced alpha diversity indices.^20^

In CC specifically, Valentova et al were able to highlight increased RVSD, with more marked gut hypoperfusion and bowel wall oedema; it was hypothesised that this could result in more exacerbated shifts to GM composition, amplifying dysbiosis beyond that seen in HF patients without cachexia.^7^ ^,^ ^22^ In our study, both *Bacteroides* and *Ruminococcus gnavus* were significant drivers of our observed beta diversity changes (Figure 2), and follow trends described in other studies in which alterations to these bacterial groups may occur with a more advanced HF phenotype.^20^ Interestingly, however, our secondary analysis did not yield any GM compositional changes in patients with and without RVSD. To our knowledge, this is the first time in the literature in which RVSD and the GM have been investigated and we provide initial evidence against the notion that RVSD alone solely drives GM changes in the absence of a CC diagnosis. From a potential pathogenic standpoint, some of the GM components responsible for the beta diversity findings have been implicated in altering local and systemic inflammatory responses. *Ruminococcae* have a recognized role in producing metabolites able to exert anti-inflammatory effects, an important example being the short-chain fatty acid (SCFA) butyrate, which helps in the maintenance of the physiologically intact blood-gut barrier alongside the suppression of pro-inflammatory T-cell responses.^23^ Studies conducted in human and animal-derived models of cancer recognize the role of dysbiosis in non-HF cancer cachexia, in which alterations to a healthy GM (notably to bacterial communities involved in the production of SCFAs such as *Ruminococcae* and *Lachnospiraceae*) may potentiate disease pathogenesis.^24^ ^,^ ^25^ Such changes may translate to overall reductions in levels of SCFA in cachexia; one study involving 107 patients with cachexia secondary to pancreatic malignancy demonstrated reductions in all major SCFA groups.^26^ Given the well-recognized inflammatory component of the CC specifically, dysbiosis could therefore potentiate CC pathogenesis through alteration to gut metabolites akin to cachexia in malignancy, highlighting a key area of future research interest.

Our secondary analyses revealed taxonomic changes across the HF spectrum and HC groups. *Streptococcaceae spp.* were significantly enriched in AHF patients when compared to HCs. (Table 2) Enrichment of *Streptococcaceae* is consistent with previously observed patterns of dysbiosis in HF. A meta-analysis of GM studies across the HF spectrum demonstrated elevated relative abundances of *Streptococcaceae* and associated *Streptococci spp*.^27^ Increased *Streptococcaceae* abundance has been associated with gut mucosal immune activation and higher intestinal permeability.^28^ Additional literature studies report upregulation of aerotolerant taxa, such as *Streptococcus*, in HF, supporting the notion that HF-related gut hypoperfusion and oedema may create an environment that promotes the proliferation of these organisms, further exacerbating proinflammatory signaling overall.^29^ In addition, *Alistipes* was more abundant with an advancing HF phenotype, enriched in AHF when compared to HF (Table 2). This remains consistent with recent systematic review and meta-analysis data observing GM changes across HF populations, albeit mechanistic links with regard to *Alistipes* and HF pathology remains elusive.^30^ Similar findings were observed with regard to Family XIII AD3011 group (*Peptostreptococcales* order), which was elevated in both AHF groups when compared to HF, and HF versus HCs (Table 2). From a mechanistic standpoint, *Peptostreptococcalae* have been associated with elevated trimethylamine *N*-oxide levels, a metabolite directly linked with HF progression and responsible for myocardial changes at the cellular level that may accelerate left ventricular dysfunction.^31^ Depletion of both *Bacteroides* and *Parasutterella* in the HF cohort, compared to HCs, was also observed, which is consistent with the current literature understanding that its relative reduction is reflective of negative overall changes to a normal, healthy GM environment.^32-34^ Finally, *Butyricimonas* was enriched in the HF cohort, relative to HCs; current evidence supports the notion that such butyrate-producing bacteria may be elevated in a compensatory attempt to reduce gut-induced inflammation post-myocardial dysfunction.^35^ ^,^ ^36^

## Limitations

Whilst this study is the first of its kind, we acknowledge some limitations worthy of consideration. Firstly, the sample size of CC/UWL-AHF patients was small, relative to the ROC; given the overall subtle changes in beta diversity and lack of overall GM compositional changes in this group, a repeat study in a larger cohort of patients with CC is pertinent. The majority of patients in this study were Caucasian men, and given the global diversity within HF, a more representative patient group would be prudent for future studies. Given the importance of gut metabolites in modulating inflammation, alongside growing evidence of their involvement in non-HF cachexia, incorporation of GM changes with metabolite data would be pertinent. Finally, we did not account for dietary patterns in this study, an important cofounder in influencing GM composition.

## Conclusion

In conclusion, this study is the first of its kind to investigate the potential pathogenic role of the GM in CC. We observed a trend towards altered microbial community structure in CC/UWL-AHF patients, suggesting that the microbiome may contribute to the complex host–metabolic interactions underlying cardiac cachexia. A future study integrating microbial composition with metabolites in a larger cohort of CC patients is warranted.

## Data Availability

The clinical data generated in this study cannot be shared owing to patient confidentiality and ethical approval constraints. The anonymized sequencing data are available in the EMBL-EBI European Nucleotide Archive (ENA) under accession number PRJEB105609.
